# Optimizing Engagement in an Online Dietary Intervention for Depression (My Food & Mood Version 3.0): Cohort Study

**DOI:** 10.2196/24871

**Published:** 2021-03-31

**Authors:** Claire Louise Young, Mohammadreza Mohebbi, Heidi M Staudacher, Frances Kay-Lambkin, Michael Berk, Felice Nellie Jacka, Adrienne O'Neil

**Affiliations:** 1 Food & Mood Centre Institute for Mental and Physical Health and Clinical Translation Deakin University Geelong, Victoria Australia; 2 Biostatistics Unit Faculty of Health Deakin University Geelong Australia; 3 Priority Research Centre for Brain and Mental Health Faculty of Health and Medicine The University of Newcastle Callaghan, New South Wales Australia; 4 Institute for Mental and Physical Health and Clinical Translation Deakin University Geelong, Victoria Australia; 5 Centre for Adolescent Health Murdoch Children’s Research Institute Parkville, Victoria Australia; 6 Black Dog Institute Syndey, New South Wales Australia; 7 James Cook University Queensland Australia

**Keywords:** online intervention, nutritional psychiatry, depression, low mood, dietary intervention, eHealth, mHealth, dietary intervention, engagement, nonusage attrition

## Abstract

**Background:**

Online interventions can be a cost-effective and efficient way to deliver programs to large numbers of people regardless of geographic location. However, attrition in web-based interventions is often an issue. Developing ways to keep participants engaged is important for ensuring validity and limiting potential biases. We developed a web-based dietary intervention as part of The My Food & Mood study which aimed to optimize ways to engage participants with low mood or depressive symptoms to promote dietary behavior change. Different versions of the My Food & Mood program were tested during optimization. Iterations were developed based on user feedback and usage analysis.

**Objective:**

The purpose of this study was to compare engagement and nonusage attrition across 4 program iterations—which differed by platform format, delivery mode, and activity type—to create an optimized version.

**Methods:**

Each program version contained modular videos with key activities with respect to implementing behavior change techniques of equivalent levels of required participation and length: version 1.0, desktop program and smartphone app; version 2.1, desktop or smartphone program; version 2.2, desktop program; and version 3.0, smartphone app. Adults with PHQ-8 scores of 5 or greater were recruited online and assigned to 1 of the 4 versions. Participants were asked to use the program for 8 weeks and complete measures at weeks 4 and 8. Engagement data were collected from the web-based platform system logs and customized reports. Cox regression survival analysis examined nonusage attrition and Kruskal-Wallis tests compared engagement across each cohort.

**Results:**

A total of 614 adults participated. Kruskal-Wallis tests showed significant differences across the 4 cohorts in all engagement measures. The smartphone app (version 3.0) had the greatest engagement as measured by weeks engaged, total usage time, total time key activities, number of active sessions, percentage of activities completed against protocol, goals completed, and percentage of videos watched. Cox regression multivariate survival analysis showed referral from a health practitioner (hazard ratio [HR] 0.344, *P*=.001) and greater proficiency with computers (HR 0.796, *P*=.049) reduced the risk of nonusage attrition. Computer confidence was associated with an increased risk of nonusage attrition.

**Conclusions:**

My Food & Mood version 3.0, a dietary intervention delivered via smartphone app with self-monitoring tools for diet quality and mood monitoring, was the version with greatest engagement in a population with low mood or depression. The iterative design techniques employed and analysis of feedback from participants resulted in a program that achieved lower rates of nonusage attrition and higher rates of intensity of use.

## Introduction

### Background

Depression is a common and debilitating mental illness that is estimated to affect 4.4% of the world’s population [1]. There is a well-documented and significant treatment gap attributable to lack of knowledge, stigma [2], and poor access [3]. Even with full provision of established psychological and pharmacological treatments, it has been estimated that only 50% of the burden of the disease is addressed [4]. The symptomatology of depression impairs function in daily life and may also impair an individual’s ability to adhere to psychological and pharmacological treatment. Subsyndromal depression is also common and causes similar impairment and impact [5]. Individuals with subsyndromal depression are at increased risk of developing clinical depression. These findings highlight an urgent need for novel and accessible treatment and prevention strategies at the population level.

Nutritional psychiatry is an emerging field of research that could potentially provide such strategies. This field investigates the relationship between diet and mental health [6] and has produced substantial observational evidence that diet quality is associated with the risk of depression [7-9]. This evidence is now supported by intervention studies [10-12] showing that intervening to improve diet quality can improve depressive symptoms. Two systematic reviews, one with meta-analysis [13,14], reported that dietary interventions are efficacious adjunctive treatments for reducing depressive symptoms. It is thought that diet quality mediates the systematic inflammation characteristic of depression by influencing the immune system response through the gut microbiota [15]; however, in order to determine if these findings can translate into accessible population-level treatment and prevention strategies, further large-scale trials are required, using platforms capable of widespread and cost-effective translation.

A digital dietary intervention for people experiencing depressive symptoms has the potential to be large scale and attract people who may not seek treatment through traditional channels. There is a substantial body of evidence supporting digital delivery of psychological interventions for depression as an accessible and feasible way to reach this population [16-18]. Digital interventions have the potential to reach isolated or resource-poor populations, can be used at a time and place convenient to the user, and provide anonymity to overcome stigma; they are likely to appeal to the segment of the population experiencing depressive symptoms that is reluctant to or unable to seek help through traditional channels. As such, a web-based dietary intervention—eHealth or mobile health (mHealth)—could be a cost-effective and scalable way to further test dietary interventions in depression. Both eHealth and mHealth dietary interventions have been shown to be comparable in effectiveness, with low to medium effect sizes [19]; however, it is important that these interventions are developed with the target population to ensure barriers to use and change are addressed in intervention design. It is also important that potential disparities to access (eg, eHealth literacy, access to technology) are addressed in dissemination strategies.

However, despite the promise of these technologies, literature suggests that they have not yet been successfully harnessed to achieve meaningful and sustained health behavior change [20,21]. In order to reach their potential, digital interventions need to address the issues of limited uptake, engagement, adherence, and high attrition rates [22,23]. Attrition rates greater than 50% are not uncommon in both eHealth and mHealth programs [24-27]. In addition, there are inconsistent approaches to the conceptualization of engagement and its measurement in the literature [28,29]. A paper reporting a recent study [28] into understanding and promoting effective engagement with digital behavior change interventions notes conceptualization should account for user experiences of the technology as well as social and therapeutic contexts and proposes that measurement of engagement should include qualitative feedback, self-report questionnaires, and logs of system usage data.

Traditionally, engagement in web-based interventions has been measured by content access measures, such as how many times a user logs into the intervention site or how many pages of intervention content are accessed [30,31]. While these are useful indicators of activity, they are not able to detail if the user has had sufficient exposure to the program or if exposure has been in a manner appropriate to digest the materials presented or adequately complete assigned tasks. If a user accesses all the content but does not complete the required assessments for the intervention and is therefore considered a dropout, there are few conclusions that can be drawn as to why. However, if analytics highlight that the user simply clicked through the content and did not spend sufficient time on the site to properly consume the intervention content, then their experience of the intervention and actual level of engagement can be better understood. These types of measurements (examining time versus access or analysis of interaction with intervention features such as time spent watching video content, drop-off points for video content, time spent on forums, or messaging within the system) are defined as intensity-of-use measures [31]. The e-CONSORT guidelines [31] recommend reporting both usage measures and intensity-of-use measures for all web-based interventions, in order to improve both the quality of publications and analysis of engagement and user interaction with these types of interventions. In addition, analyzing these types of data against intervention outcomes will enable robust estimates of effective engagement [21].

Understanding how participants use digital interventions has been identified as key to understanding the issues of limited uptake, engagement, adherence, and high attrition rates [20]. Analyzing nonusage attrition, as defined by Eysenbach [30], is also important for understanding these issues. This has prompted more research into understanding usage and nonusage attrition in digital interventions [32,33]. Understanding how users interact (usage and nonusage) with a digital intervention can help to better measure adherence and intervention effectiveness [23]. In addition, the trajectory of symptoms and the experience of depression itself may impede participants’ abilities to adhere to or engage with a digital intervention. Understanding usage patterns and nonusage attrition might help guide future intervention designs that are more accommodating of the impact of symptoms or more engaging for the target population.

The My Food and Mood study aimed to develop and optimize a digital (eHealth and mHealth) dietary intervention for people experiencing depressive (including subclinical) symptoms. From 2017 to 2019, the My Food & Mood study developed an initial version of the program (My Food & Mood Program version 1.0) guided by evidence from nutritional psychiatry, behavior change, expert input, and consumer input. Using principles of user-centered design, this study iteratively optimized the program based on qualitative feedback, self-report questionnaires, and analysis of both usage and intensity of use engagement measures. Each version of the program was trialed by a separate cohort of the target population. During the course of the optimization phase, 3 subsequent versions of the program (My Food & Mood Program version 2.1, version 2.2, and version 3.0) were produced and trialed. This study is an analysis of the quantitative engagement data collected during the My Food and Mood study.

### Objectives

The primary aim was to analyze patterns of usage across all 4 cohorts of the My Food and Mood study to understand quantitative differences in engagement across each version and to determine which version of the program had the highest rates of user engagement and lowest rates of nonusage attrition. The secondary aim was to identify factors that predicted active usage and nonusage attrition across all cohorts.

## Methods

### Study Design: My Food & Mood Study

The My Food & Mood study was conducted from 2017 to 2019. The study design was guided by the Information Systems in Research framework [34]. The study initially involved the development and testing of a web-based dietary intervention program for people with depressive (including subclinical) symptoms. This resulted in the first version of the program (My Food & Mood version 1.0) which was trialed by cohort 1 from October 2018 to March 2019 (recruitment round 1). Subsequent phases of optimization were run with the second (version 2.1), third (version 2.2), and fourth (version 3.0) versions of the programs from June 2019 to January 2020 (recruitment round 2) and trialed by separate cohorts (2.1, 2.2, and 3.0, respectively). Design iterations informed by feedback and analysis of engagement from preceding cohorts aimed to optimize the program. Standard software development version control methods were employed to manage each iteration. Participants in each cohort had access to their respective version of the program for an 8-week period. Feasibility analysis for the optimized version has been published elsewhere [35]. This analysis examined the quantitative engagement measures collected during the My Food & Mood study to look at usage and nonusage attrition across each version of the program.

### Participants

Participants were recruited by targeted email campaigns, online advertising, and social media posts. Campaign emails were sent to members of the Food & Mood Centre’s potential participants database and to members of the Community and Research Network run by Innovation in Mental and Physical Health and Clinical Treatment Strategic Research Centre Strategic Research Centre. The program was also advertised by Beyond Blue to members of Blue Voices, a community with lived experience who contribute to the development of mental health services, policy, and programs [36]. Targeted advertisements were placed on Facebook ads, and social media posts were disseminated through the Food & Mood Centre’s channels. Printed flyers were distributed at Barwon Health’s acute mental health inpatient clinic, and the program was advertised during community presentations conducted by the Food & Mood Centre researchers.

Screening was performed online via the recruitment website [37]. Recruitment material and the recruitment website were branded with the Food & Mood Centre, Innovation in Mental and Physical Health and Clinical Treatment Strategic Research Centre, and Deakin University logos. Participation was voluntary, and all participants provided digital consent prior to commencement. The study was conducted in accordance with the National Statement on the Ethical Conduct of Research and the protocol was approved by Deakin University Faculty of Health’s Ethics Committee (reference 14/SW/1127).

### Inclusion and Exclusion Criteria

The screening survey included demographic questions, questions about access and use of technology, and questions about dietary autonomy. Computer proficiency was evaluated using the Computer Proficiency Questionnaire (CPQ-12) [38], and screening for possible eating disorders was conducted using the Sick, Control, One, Fat, and Food (SCOFF) questionnaire [39]. The 8-item Patient Health Questionnaire (PHQ-8) was used to screen for depressive symptoms. [40] In recruitment round 1, eligible participants completed the Simple Dietary Questionnaire (SDQ) as part of the baseline questionnaires. In recruitment round 2, the Mediterranean Diet Adherence Screener (MEDAS) assessed level of adherence to a Mediterranean diet [41].

Individuals were eligible for the study if they were aged over 18 years and reported current depressive symptoms (PHQ-8 score ≥5). Individuals were excluded if they did not have access to the internet, a computer, or smartphone; had low computer literacy (CPQ-12 score <10 ); had limited English literacy; were not able to follow a different diet (no diet autonomy); and if there was risk of eating disorder (SCOFF score ≥2). In recruitment round 1, participants were excluded if they were not located in Australia or the United States. This additional criterion was due to the available versions of the diet recall tool that was used in this part of the trial. In recruitment round 2, individuals were also excluded if they already followed a high-quality diet (MEDAS score >11).

### Interventions

#### Overview

All 4 versions of the program addressed the relationship between diet and mental health. Advice provided in version 1.0 was for a diet for good gut health; this focused on increasing fiber intake and decreasing discretionary food intake and was aligned to the Australian Dietary Guidelines [42]. It included food recommendations based on traditional dietary patterns such as the Mediterranean diet. The dietary advice was updated for versions 2.1, 2.2, and 3.0 based on user feedback and expert analysis of the outcomes from cohort 1. The advice for these versions was for a modified Mediterranean style diet that aligned to the Australian Dietary Guidelines [42]. The dietary advice was delivered via video, in question and answer format, and filmed using Food & Mood Centre researchers. All versions implemented the same behavior change techniques. The key behavior change techniques and their implementation are summarized in Multimedia Appendix 1. Each of the programs required equivalent levels of participation for the key behavior change activities (key activities). Key activities for each program and the expected duration required are listed in Multimedia Appendix 2. The versions differed in delivery platforms and formats (version 2.1: web-based or smartphone program; version 2.2: web-based–only program; version 3.0: smartphone app only). The web-based programs were built in Moodle 3.6 (developed by Martin Dougiamas) and were accessed via personal computer through a supported browser. Version 2.1 could also be accessed through the Moodle app. The smartphone apps were custom developed using Corona Labs (graphics depicting the user interfaces for each version are shown in Multimedia Appendix 3).

#### My Food & Mood Version 1

Version 1 was a 6-module web-based program and accompanying smartphone monitoring app. Each module contained an educational video and 6 reinforcement activities (game, quiz, additional reading, shopping list, recipe, and goal setting). The smartphone app enabled self-monitoring of diet and mood with simple graphical inputs and produced a graph of daily food and mood scores. Participants had 8 weeks to complete the 6 modules. Participants were advised to use the smartphone app daily from week 1.

#### My Food & Mood Version 2.1

Version 2.1 was a web-based program that delivered the intervention content as 16 discrete short modules. The program was optimized for use on a desktop but could also be accessed on a smartphone. Each module contained a video explaining an aspect of the Mediterranean diet and an activity or a short quiz about the video content. The program also included a web-based activity to self-monitor diet. The videos were between 1.5 to 3 minutes in length. Participants could work through the videos and activities at their own pace over the 8-week period.

#### My Food & Mood Version 2.2

My Food & Mood version 2.2 was a web-based–only program that delivered the intervention content in a week-by-week format. The program also included a web-based activity to self-monitor diet. The first 2 weeks presented the intervention video content as compiled modules. Modules 1 to 8 were presented in week 1 and modules 9 to 16 in week 2. The subsequent 6 weeks of the intervention presented recipes and goal setting activities.

#### My Food & Mood Version 3.0

My Food & Mood Version 3.0 was a custom-built smartphone app. The intervention content was presented as links with the content divided as per version 2.2 (Modules 1-8, Modules 9-16). The app also contained self-monitoring tools for diet, mood, and lifestyle as well as tools for goal setting and food shopping. Participants could see their progress against the ModiMed Diet score [43] on the progress page, which reflected improvement needed (with respect to food groups) to achieve higher diet quality.

### Measures

#### Sample Characteristics

Participant characteristics were derived from the screening survey. BMI was calculated from self-reported height and weight. Socioeconomic index and remoteness area classification were coded from Australian Bureau of Statistics datasets [44,45] for Australians who supplied postcodes. Computer confidence was self-rated on a Likert scale between not *confident at all* to *confident* and computer skill level was also self-rated on a Likert scale between *never used* to *highly skilled*.

#### Diet Quality

Diet quality was measured at baseline, week 4, and week 8 using the validated MEDAS [46,47] and the SDQ (Parletta N, unpublished). The MEDAS is a 14-item scale with a maximum score of 14. It has acceptable accuracy and reliability for assessing adherence to a Mediterranean diet [41]. The SDQ is a 27-item food frequency questionnaire, based on the Australian Dietary Guidelines, that is suitable for self-report and simple to complete. Its score range is 0 to 100 and it has been validated against 24-hour recall and demonstrated moderate validity correlations (*r*=0.42 to 0.57; Parletta N, unpublished data). A combined baseline diet quality score (MEDAS Rescored) was calculated for both the MEDAS and SDQ responses; this included responses to overlapping questions from the 2 instruments. Diet quality question mapping and scoring is shown in Multimedia Appendix 4.

#### Depressive Symptoms

Depressive symptoms were measured at baseline, week 4, and week 8 using the PHQ-8. PHQ-8 is reliable and valid 8-item assessment of depressive symptoms with a score ranging from 0 to 24. PHQ-8 has been shown to be suited to self-reporting [48]. Baseline depressive symptom severity was calculated from PHQ-8 scores (mild, score 5-9; moderate, score, 10-14; moderately severe, score 15-19; and severe, score >20) [49].

#### Engagement

Engagement was represented by duration, frequency, and intensity-of-use measures; 8 engagement measures were calculated from database entries, timestamped event logging from active sessions, and a custom script tracking the duration of videos watched. An active session was recorded any time the participant logged into the desktop app or opened the smartphone app and accessed content or made data entries. The measures were (1) *weeks engaged*: duration of program use in weeks calculated by subtracting the date and time of the last active session from the date and time of the first active session; (2) *total usage time*: calculated from the sum total time for all active sessions; (3) *total time key activities*: calculated from the sum total time of active sessions for key activities; (4) *number of active sessions*: total count of active sessions; (5) *average time per session*: mean total time of active sessions; (6) *per protocol percentage*: total number of completed key activities divided by total number of key activities; (7) *goals completed*: number of goals set and marked as complete; and (8) *percentage videos watched*: maximum duration watched for each video divided by video duration.

#### Nonusage Attrition

Weeks engaged, per protocol percentage, and number of active sessions were used as indicators of nonusage attrition. Participants were coded as *nonusage attrition observed* if there were less than 4 active sessions recorded over the 8-week period and if they had completed less than 90% of the key activities (per protocol percentage) for their program by their last active session. (These requirements were based on the optimization protocol and the number of sessions required to complete the key activities.) Participants who recorded more active sessions and had completed more than 90% of the key activities (per protocol percentage) were coded as *nonusage attrition not observed*.

An active participant was defined as a participant who used their allocated program. Use was determined as those who logged in to the desktop program at least once (version 1.0, 2.1, and 2.2) or downloaded and logged into the smartphone app (version 1.0 and 3.0).

### Statistical Analysis

The sample size required to detect an improvement in engagement, calculated from time required per activity, was based on the initial program design (My Food & Mood version 1.0). In this program, the 6 modules required 15 to 20 minutes engagement time. Assuming mean 90 (SD 25) minutes for engagement time, a sample size of 100 participants per cohort, and type I error of .05, the study had 80% power to detect 10 minutes or greater improvement in mean engagement time across each version of the program. This is equivalent to a moderate effect size of 0.4.

Nonusage attrition rate across weeks engaged was analyzed using Cox regression multivariate survival analysis, with weeks engaged and the coded survival variable. Univariate analyses were conducted on baseline characteristic variables of interest to select covariates for the model. Selected characteristic variables were age, gender, recruitment source, BMI, education, employment, computer skills, computer confidence, baseline mood, and diet quality. Cox regression multivariate survival analysis was repeated including only active participants. Intervention engagement was evaluated by comparing median engagement time and intensity of use outcomes (weeks engaged, total usage time, total time key activities, number of log-ins, average time per log-in, per protocol percentage, goals completed, percentage videos watched) between the 4 cohorts using Kruskal-Wallis *H* tests.

## Results

### General

A total of 614 adults were recruited online into the 4 cohorts at the 2 time points. Participants were predominantly female (536/614, 87.3%) and from Australia (443/614, 70.5%). Of those who supplied a valid Australian postcode (304/614, 49.5%), 79.3% (241/304) were from major cities, 20.1% (61/304) were from regional areas, and 0.7% (2/304) from remote areas. Most participants had a university education and lived in higher-ranked socioeconomic index areas. Table 1 presents the demographic characteristics of each cohort. There was a significant difference in age (χ^2^=12.295, *P*=.006) baseline PHQ-8 (χ^2^=11.323, *P*=.01), and baseline MEDAS Rescored (χ^2^=26.093, *P*<.001) across cohorts. Consort diagrams for each recruitment round are provided in Multimedia Appendix 5.

**Table 1:** Characteristics of participants from each of the cohorts.

Variable		Cohort 1 (n=156)	Cohort 2.1 (n=154)	Cohort 2.2 (n=151)	Cohort 3 (n=153)
**Gender, n (%)**					
	Male	34 (21.8)	14 (9.1)	19 (12.6)	28 (18.3)
	Female	122 (78.2)	140 (90.9)	132 (87.4)	125 (81.7)
Age, median (quartile 1, quartile 3)		40 (32, 49)	37 (30, 45)	41 (34, 49)	42.5 (33, 50)
BMI, mean (SD)		26.41 (5.68)	25.85 (7.19)	26.09 (6.05)	26.28 (6.11)
Depressive symptoms (Mood PHQ-8^a^), median (quartile 1, quartile 3)		8 (6, 12)	10 (6, 12)	10 (7, 15)	10 (7, 14)
Taking antidepressants, n (%)		50 (23.4)	49 (22.9)	59 (27.6)	56 (26.2)
Diet quality, MEDAS^b^ (Rescored), median (quartile 1, quartile 3)		3 (2, 4)	2 (1, 3)	2 (1, 3)	2 (2,3)
Socioeconomic index, median (quartile 1, quartile 3)		8 (6, 9)	9 (6, 9)	8 (5, 9)	7 (5, 9)
**Recruitment referral source, n (%)**					
	Facebook	67 (43.1)	64 (41.5)	65 (42.9)	66 (43.0)
	Instagram	11 (7.0)	11 (6.9)	10 (6.3)	11 (7.1)
	Twitter	19 (12.0)	20 (13.1)	18 (11.6)	19 (12.2)
	The Food & Mood Centre	12 (7.9)	12 (8.1)	13 (8.3)	12 (7.8)
	Health Practitioner	29 (18.3)	26 (17.2)	26 (16.9)	26 (17.3)
	Family	9 (6.0)	11 (7.1)	10 (6.7)	9 (6.1)
	Friend	7 (4.5)	8 (5.1)	8 (5.2)	7 (4.3)
	Other	2 (1.2)	2 (1.0)	3 (2.1)	3 (2.2)
**Education, n (%)**					
	Less than high school	2 (1.5)	2 (1.3)	3 (2.0)	4 (2.6)
	High school graduate	9 (6.8)	7 (4.5)	9 (6.0)	11 (7.3)
	Some college	18 (13.5)	20 (13.0)	17 (11.4)	23 (15.3)
	2-year degree	8 (6.0)	12 (7.8)	13 (8.7)	7 (4.7)
	4-year degree	41 (30.8)	36 (23.4)	23 (15.4)	30 (20.0)
	Professional degree	47 (35.3)	48 (31.2)	45 (30.2)	38 (25.3)
	Doctorate	8 (6.9)	7 (4.5)	5 (3.4)	7 (4.7)
**Employment, n (%)**					
	Employed full time	69 (44.5)	66 (42.9)	52 (34.9)	65 (43.3)
	Employed part time	50 (32.2)	46 (29.9)	55 (36.9)	45 (30.0)
	Unemployed (looking)	5 (3.2)	6 (3.9)	8 (5.4)	5 (3.3)
	Unemployed (not looking)	6 (3.9)	6 (3.9)	9 (6.0)	13 (8.7)
	Retired	2 (1.3)	3 (1.9)	4 (2.7)	6 (4.0)
	Student	6 (3.9)	4 (2.6)	5 (3.4)	6 (4.0)
	Disabled	17 (11.0)	23 (14.9)	16 (10.7)	10 (6.7)
**Computer skill level, n (%)**					
	Never used	0 (0)	0 (0)	0 (0)	0 (0)
	Beginner	1 (0.7)	4 (2.6)	0 (0.0)	2 (1.3)
	Competent	144 (97.3)	74 (48.1)	84 (56.4)	75 (50.0)
	Highly skilled	3 (2.0)	76 (49.4)	65 (43.4)	73 (48.7)
**Computer confidence, n (%)**					
	Not confident at all	0 (0)	0 (0)	0 (0)	0 (0)
	I usually need help	0 (0)	1 (0.6)	0 (0)	2 (1.3)
	It depends on the task	22 (14.9)	18 (11.7)	17 (11.4)	16 (10.7)
	Confident	126 (85.1)	135 (87.7)	132 (88.6)	132 (88.0)

^a^PHQ-8: Patient Health Questionnaire.

^b^MEDAS: Mediterranean Diet Adherence Screener.

### Engagement

**Figure 1:**
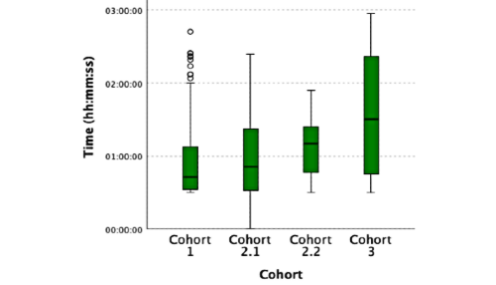
Total time key activities.

**Figure 2:**
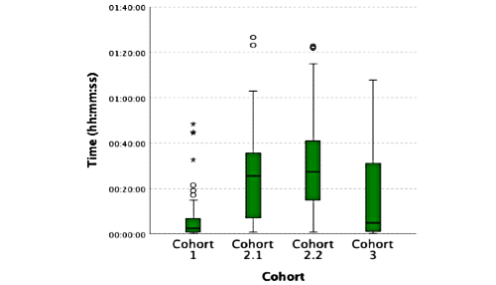
Average duration per session.

**Figure 3:**
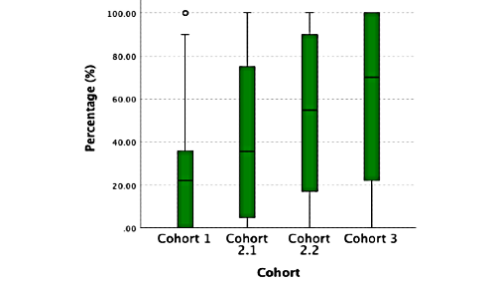
Percentage watched videos.

**Figure 4:**
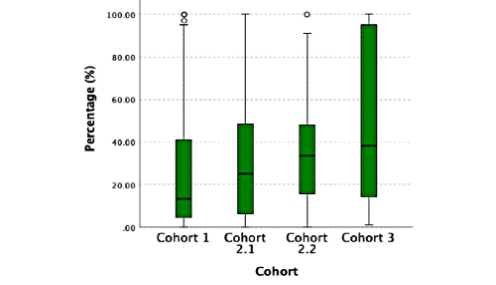
Per protocol percentage.

Table 2 presents the comparisons for engagement measures across cohorts. Cohort 3 received the highest mean rank score for a majority of the engagement measures, including weeks engaged, total usage time, total time key activities, number of sessions, percentage of activities completed against protocol, goals completed, and percentage videos watched (Figures 1-4).

**Table 2:** Engagement metric comparisons across cohorts.

Measures		Kruskal Wallis test statistics			Cohorts, median (95% CI)			
		n (*df*)	*H*	*P* value	Cohort 1	Cohort 2.1	Cohort 2.2	Cohort 3
**Usage measures**								
	Weeks engaged	424 (3)	12.573	.006	1.1 (0.6, 2.2)	1.4 (0.7, 2.1)	2.7 (1.2, 3.7)	3.6 (2.1, 4.1)
	Total usage (hours:minutes:seconds)	424 (3)	22.077	<.001	1:15:20 (0:48:29, 1:43:59)	1:28:30 (1:06:23, 1:55:00)	1:35:16 (1:22:21, 1:50:12)	1:52:15 (1:28:32, 2:22:55)
	Total time key activities (hours:minutes:seconds)	424 (3)	48.392	<.001	0:42:51 (0:40:23, 0:49:19)	0:51:24 (0:45:00, 0:59:09)	1:10:12 (0:58:49, 1:22:05)	1:30:01 (0:52:00, 1:48:00)
	Goals completed, n	424 (3)	30.426	<.001	1 (0, 2)	1 (1, 2)	2 (0, 3)	3 (2, 4)
	Active sessions, n	424 (3)	61.208	<.001	15 (6, 20)	3 (1, 4)	3 (3, 4)	30 (17, 24)
**Intensity of use measures**								
	Average duration per session (hours:minutes:seconds)	424 (3)	77.057	<.001	0:02:25 (0:01:28, 0:05:06)	0:21:41 (0:05:01, 0:30:00)	0:27:27 (0:24:58, 0:31:11)	0:04:55 (0:03:25, 0:08:22)
	Per protocol percentage	424 (3)	28.527	<.001	12.7 (11.4, 16.9)	25.3 (14.4, 34.4)	33.5 (27.7, 43.0)	38.4 (25.5, 65.6)
	Percentage videos watched	424 (3)	39.164	<.001	22.0 (13.0, 26.0)	35.5 (23.0, 48.0)	55.0 (46.0, 70.0)	70.00 (47.5, 83.5)

### Nonusage Attrition

**Figure 5:**
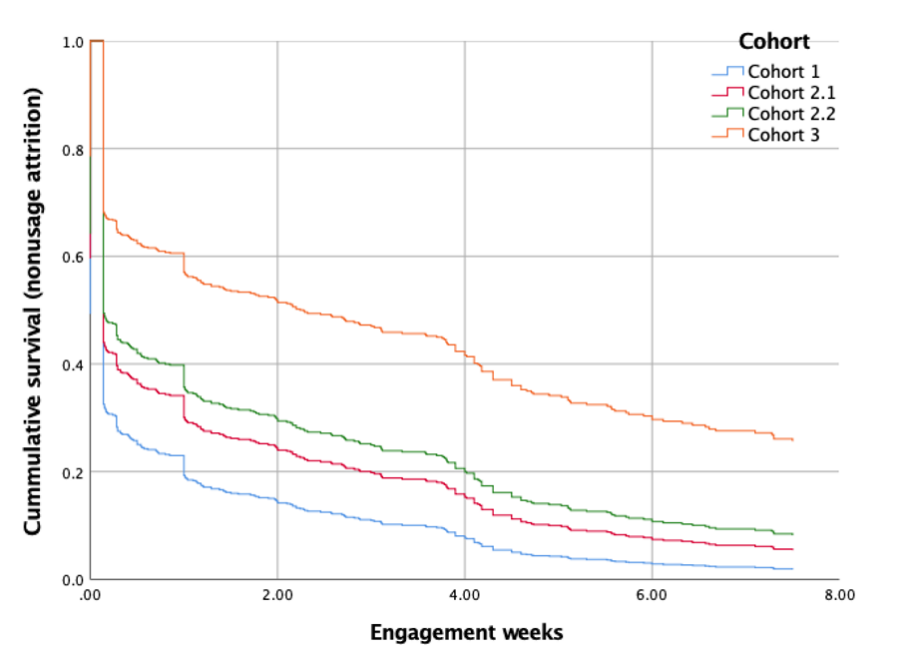
Survival curve (Cox regression) for nonusage attrition versus weeks engaged across all cohorts of the My Food & Mood Program.

Survival curves for each cohort from the Cox regression survival analysis are shown in Figure 5. Cohort 3 (My Food & Mood Program version 3.0) had the lowest rates of nonusage attrition versus weeks engaged. All survival curves show a drop in cumulative survival (the percentage of participants still using the intervention) after 1 week. Significant predictors of active usage were referral from a health practitioner (hazard ratio [HR] 0.344, 95% CI 0.179-0.660, *P*=.001) and high computer skills (HR 0.796, 95% CI 0.634-0.999, *P*=.049). High computer confidence was a significant predictor of nonusage attrition (HR 1.511, 95% CI 1.111-2.056, *P*=.009). No other variables were significant predictors of nonusage attrition.

When including only active participants, referral from a health practitioner remained a significant predictor of active usage across all versions of the intervention (HR 0.277, 95% CI 0.123-0.626, *P*=.002); however; computer skills rated as highly skilled did not (HR 0.799, 95% CI 0.597-1.068, *P*=.13). In this model, age was also a significant predictor of active usage (HR 0.987, 95% CI 0.974-1.00, *P*=.047). Computer confidence rated as confident remained a significant predictor of nonusage attrition (HR 1.604, 95% CI 1.065-2.437, *P*=.03).

## Discussion

### Principal Findings

Of 4 versions of a digital-based dietary program in individuals with low mood or depression, a smartphone app version was associated with greatest engagement and the lowest levels of nonusage attrition. These results indicate that the optimization process led to the development of a program that had improved uptake in the target population. The analysis also showed that nonusage attrition was minimized if participants were referred to the program by a health practitioner or if they rated their computer skills high. Somewhat paradoxically, however, high computer confidence was a significant predictor of nonusage attrition. In active users (those who recorded duration using the programs), we found that older adults were more likely to continue to use their allocated program. There were no relationships found between nonusage attrition and baseline depression symptoms or diet quality.

Analysis of quantitative engagement measures showed that the optimization of the program resulted in the latest version of the program (ie, the smartphone app) being a more acceptable version compared to all other versions. Participants who used this version of the program completed more activities, spent more time using the app, completed more goals and watched more of the intervention video content. Version 3.0 required that participants log daily food and mood entries and also prompted them to do so. This more regular interaction did not increase participant burden but appeared to improve usage and engagement. This is contrary to evidence from the field that suggests continually requiring data entry from participants introduces burden that may gradually erode the intervention’s effectiveness [50]. Literature also suggests that participants find it difficult to maintain routine self-monitoring over time [51]. It is possible that the consistent prompts and ability to reflect on their daily diet quality and mood may have had a reinforcing effect in this population.

The versions that directed intervention participation over the course of the 8 weeks (versions 2.2 and 3.0) had higher rates of engagement and completion of activities. These versions directed the users to complete the videos in the first 2 weeks and perform a series of behavior change activities in the following 6 weeks. This structured approach seemed to be preferred by participants. Even though this required all videos to be watched in the first 2 weeks, participants in cohorts 2.2 and 3.0 watched a greater percentage of the intervention videos compared to cohorts 1.0 and 2.1. The videos in version 1.0 were also of a longer format than and with different content to those in subsequent versions; however, the videos in version 2.1 were the same as those in version 2.2 and version 3.0. These results suggest that the structure and direction was more important for prompting engagement and completion than the length of the videos.

There were also more active participants (ie, participants who accessed the program at least once) for the version 3.0 program. This was despite the additional step required to access this version. Participants allocated to this version had to install the app to their smartphone prior to logging in, whereas participants using the desktop programs only needed to click a link to access their version. With ubiquitous use of mobile phones across the globe, this is a promising finding for the design and dissemination of dietary interventions.

While many researchers have investigated predictors of adherence and attrition, there is little consensus as to which defining characteristics might predict active engagement with a digital intervention [26,32]. Analysis into predictors of nonusage attrition from psychological web-based interventions for depression have reported lower baseline rates of depression, younger and older age, low levels of education, and poorer knowledge of psychological treatments as predictors [52,53]. Our results only concur with theirs for age as a significant predictor of active usage (HR 0.987, 95% CI 0.974-1.00, *P*=.047) when analysis nonusage attrition for active users. This result may be counterintuitive as there is a common assumption that younger users are more likely to engage with technology due to their higher levels of technology use [54]; however, there is a growing body of literature indicating older adults are more likely to remain engaged with digital interventions.

We found no relationship between baseline depressive symptoms and nonusage attrition and our subsequent feasibility analysis [35] showed that participants were able to complete the program independent of the severity of their baseline symptoms. This is concordant with findings from clinical intervention trials in nutritional psychiatry that have found dietary change was possible independent of the level of baseline depression severity. The evidence to date supports dietary improvement as a feasible and acceptable treatment strategy for people experiencing depressive symptoms.

In addition, there was also no relationship between nonusage and baseline diet quality, the primary outcome measure for the feasibility study. This finding shows participants with low diet quality and more opportunity for improvement could engage with the intervention as well as those with higher diet quality and less opportunity for improvement.

One key finding from the analysis of nonusage attrition was that participants referred to the program by a health practitioner were more likely to use the program. There are two reasons why this may have been the case. Referral by health care practitioners may have increased the perceived credibility of the program, thereby encouraging usage. Second, participants referred by health practitioners may have been identified as well-suited to this form of adjunctive treatment. Increased engagement due to these informal referral channels is a positive finding and an important consideration for the design and dissemination of future interventions of this kind. In order to increase participation and reduce the risk on nonusage attrition in future trials and treatment programs, more formal referral and recruitment processes utilizing health practitioners should be employed.

Participant’s with higher self-rated computer skills were more likely to remain actively using the program. However, if users rated their computer confidence as high, this was a significant predictor of nonusage attrition. These findings appear contradictory; we would expect confident users to encounter fewer barriers to navigating through or actively using an eHealth intervention. Participant characteristics across all cohorts showed there were much larger proportions of participants rated as confident compared to any other level. Given the common use of technology in modern society, and the limited range of options for this response the majority of participants rating themselves as confident is perhaps not surprising. It may be that additional nontechnical barriers limited progress of participants through the programs. Even if perceived confidence is high and participants are willing to engage, having inadequate skills may be one such barrier. In order to manage this, strategies to improve participants skill-level to improve nonusage attrition rates could be introduced in the initial program stages, especially focusing on the skills required to participate in the intervention.

The nonusage attrition analysis highlights the need to address attrition in the early stage of digital interventions. A large percentage of participants across all cohorts did not use the intervention (pretreatment dropouts) or only used the intervention for a short period of time, with nonusage attrition curves across all cohorts showing significant drop in usage after 1 day or within the first week. Qualitative feedback was collected during the web-based programs and follow-up surveys 4 and 8 weeks after the program [35]; however, for those who never accessed the intervention, we were unable to ascertain why this was the case.

The screening survey ended with links and log-in information to directly access the program or download the required smartphone app. This was designed to be a seamless transition to starting the programs. Analysis of participant behavior at this final screen might aid the design of future interventions. An additional prompt, given prior to closing the browser for participants who do not click on the access links, similar to notifications used by marketing websites, might improve these access numbers. In addition, the access information was also sent to participants via email. Automating follow-up of click through rates on access information emails for those yet to access the program might also be an opportunity to prompt users to log-in.

### Strengths and Limitations

The strengths of this study include the quantitative data analysis of engagement across all participants, as these data were available for the entire cohort. Moreover, active engagement of people with lived experience, including members of Beyond Blue’s Blue Voices, informed the design of the program and produced an mHealth version that was able to be used by people experiencing differing severities of depression symptoms. The study design allowed multiple internet delivery parameters to be explored. Lastly, the study met its recruitment targets such that sufficient power to explore endpoints was available.

Despite the strengths of this study, there were also limitations. The dietary advice changed between the first version and the subsequent versions of the program. The change in dietary advice to a more prescriptive Mediterranean diet resulted from relevance and rigor cycles and were defined by the Information Systems Research framework [34] that was undertaken in the optimization phase. User feedback, expert dietitian input, and review of current literature resulted in the decision to modify the dietary advice to be more prescriptive. While the dietary advice delivered in version 1.0 was not a prescriptive Mediterranean diet, as it was in the 3 subsequent versions (version 2.1, version 2.2, and version 3.0), the programs were comparable in the style, content, delivery, and equivalence of key behavior change activities required.

A large proportion of participants were based in Australia, which may limit global generalizability. In addition, we were unable to collect feedback from those who left the study without downloading the app. Without this feedback, it is difficult to address the reasons the different versions of the program were not able to capture their attention.

### Conclusions

The optimization study of the My Food & Mood program resulted in an mHealth version of a dietary intervention that had higher levels of usage and engagement than 3 previous versions of the intervention. More participants using this version completed more of the assigned activities and remained engaged, actively using the program for longer. These findings will inform and support the development of future large-scale trials aimed at further testing dietary interventions in depression.

Analysis of nonusage attrition showed that referral by a health practitioner reduced the risk of nonusage attrition. In addition, nonusage was independent of participants’ depressive symptoms or diet quality. While several researchers have investigated predictors of adherence and attrition, there is little consensus in the literature of which characteristic might predict participants actively engaging with a digital intervention [26,32]. Our findings contribute further to this discussion.

## Appendix

Multimedia Appendix 1

Multimedia Appendix 2

Multimedia Appendix 3

Multimedia Appendix 4 Diet quality question mapping and scoring.

Multimedia Appendix 5

## Abbreviations

CPQ: Computer Proficiency Questionnaire
HR: hazard ratio
MEDAS: Mediterranean Diet Adherence Screener
mHealth: mobile health
PHQ-8: 8-item Patient Health Questionnaire
SCOFF: Sick, Control, One, Fat, Food
SDQ: Simple Dietary Questionnaire
