# Supplementary material for: Optimizing Engagement in an Online Dietary Intervention for Depression (My Food & Mood Version 3.0): Cohort Study
Source: JMIR Ment Health. 2021 Mar 31;8(3):e24871. doi: 10.2196/24871 (PMC8047812; doi:10.2196/24871)
Supplement: Multimedia Appendix 1 [file mental_v8i3e24871_app1.pdf]

Table 1: My Food and Mood Versions 1.0, 2.1,2.2 & 3.0 Behaviour Change Techniques Summary

**Target Behaviour**	**Behaviour Change Techniques**	**Module Components/ Activities**			
		**V1.0**	**V2.1**	**V2.2**	**V3.0**
Knowledge Motivating Change	5.1. Information about health consequences<br>5.6. Information about emotional consequences<br>9.1. Credible source	Module Content (Video)<br>Key Messages (PDF)<br>Review Quiz 1-6<br>Reinforcement activities	Module Content (Video)<br>Key Messages (PDF)<br>Review Quiz 1<br>Review Quiz 2	Module Content (Video)<br>Key Messages (PDF)<br>Modules 1-8 Quiz<br>Modules 9-16 Quiz	Module Content (Video)<br>Key Messages (PDF)<br>Modules 1-8 Quiz<br>Modules 9-16 Quiz
Identify Motivation to change	3.3 Social Support (emotional)	Motivation to Change (Quiz)<br>Health & Lifestyle Diet<br>*Participants are given feedback about their level of motivation and directed to resources to increase motivation (if required)*	Motivation to Change (Quiz)<br>Health & Lifestyle Diet<br>*Participants are given feedback about their level of motivation and directed to resources to increase motivation (if required)*	Motivation to Change (Quiz)<br>Health & Lifestyle Diet<br>*Participants are given feedback about their level of motivation and directed to resources to increase motivation (if required)*	Motivation to Change (Quiz)<br>Health & Lifestyle Diet<br>*Participants are given feedback about their level of motivation and directed to resources to increase motivation (if required)*
Commitment to change	1.4 Problem Solving<br>1.9 Commitment	Identifying barriers to change<br>Identifying SMART Goals	Identifying SMART Goals<br>Implementing strategies to achieve SMART goals	Identifying SMART Goals<br>Implementing strategies to achieve SMART goals	Implementing strategies to achieve SMART goals<br>Shopping Lists
Performing the behaviour	6.1 Demonstration of the behaviour<br>9.1. Credible source	Module content (SMART Goals)<br>Recipes<br>Videos by Food & Mood Centre researchers	Module content (SMART Goals)<br>Recipes<br>Videos by Food & Mood Centre researchers	Module content (SMART Goals)<br>Recipes<br>Videos by Food & Mood Centre researchers	Module content (SMART Goals)<br>Recipes<br>Videos by Food & Mood Centre researchers<br>Shopping List Template
Goal Setting	1.1. Goal setting (behaviour)<br>10.4 Social Reward	Modular Goal Setting activity<br>*Guided template to set a SMART goal and prompts to complete it (All Modules)*<br><br>*Participants receive award in My Food & Mood on completion of their goal*	Modular Goal setting activities<br>*Participants guided through setting a weekly SMART goal and prompted to complete it (Modules 6, 7, 8, 9, 11, and 16)*<br>*Participants receive award in My Food & Mood on completion of their goal*	Weekly Goal setting activities<br>*Participants guided through setting a weekly SMART goal and prompted to complete it (Modules 6, 7, 8, 9, 11, and 16)*<br>*Participants receive award in My Food & Mood on completion of their goal*	Weekly Goal setting activities<br>*Guided template for SMART goal and notifications and prompts to complete them (Modules 6, 7, 8, 9, 11, and 16)*<br>*List of completed goals available under progress*

<table>
<tr><th rowspan="2">Target Behaviour</th><th rowspan="2">Behaviour Change Techniques</th><th colspan="4">Module Components/ Activities</th></tr>
<tr><th>V1.0</th><th>V2.1</th><th>V2.2</th><th>V3.0</th></tr>
<tr><td>Monitoring diet quality</td><td>2.3. Self-monitoring of behaviour</td><td>Daily Food Data Entry (Daily input)
Participants are given feedback progress toward the best score over a 7 day period.</td><td>ModiMed Diet Scoring Activity (Daily input)
Participants are given feedback progress toward the best score over a 7 day period.</td><td>ModiMed Diet Scoring Activity (Daily input)
Participants are given feedback progress toward the best score over a 7 day period.</td><td>Daily Food Data Entry (Daily input)
Participants are given feedback progress toward the best score over a 7 day period.</td></tr>
<tr><td>Knowledge
Modelling the Behaviour
Re-framing
Motivating Change</td><td>10.5 Social Incentive
2.2. Feedback on behaviour</td><td>Self-monitoring of diet and mood produces graph to encourage identification of patterns</td><td>Self-monitoring of diet results in daily score showing Mediterranean Diet adherence

Rewards are achieved based on goal setting and goal completion.
Points are also awarded based on progress through the program</td><td>Self-monitoring of diet results in daily score showing Mediterranean Diet adherence

Rewards are achieved based on goal setting and goal completion.
Points are also awarded based on progress through the program</td><td>Self-monitoring of diet results in daily heatmap showing progress with Mediterranean Diet adherence</td></tr>
</table>
