# Supplementary material for: Optimizing Engagement in an Online Dietary Intervention for Depression (My Food & Mood Version 3.0): Cohort Study
Source: JMIR Ment Health. 2021 Mar 31;8(3):e24871. doi: 10.2196/24871 (PMC8047812; doi:10.2196/24871)
Supplement: Multimedia Appendix 2 [file mental_v8i3e24871_app2.pdf]

Supplementary Material

My Food Mood Programs Key Activity Time Requirements

**My Food & Mood Version 1.0**		**My Food & Mood Version 2.1**		**My Food & Mood Version 2.2**		**My Food & Mood Version 3.0**	
Videos							
Module 1: Introduction	0:10:29	Module 1: Introduction	0:02:11	Modules 1 to 8	0:28:59	Modules 1 to 8	0:28:59
Module 2: What is good gut health?	0:06:45	Module 2: How does diet improve mental health?	0:02:25	Modules 9 to 16	0:23:41	Modules 9 to 16	0:23:41
Module 3: What is fibre?	0:11:50	Module 3: The Mediterranean Diet	0:03:49				
Module 4: Pre & Pro biotics	0:10:45	Module 4: How do I make changes to my diet?	0:03:58				
Module 5: Fats & Proteins	0:06:35	Module 5: Plant foods: fruit and vegetables	0:03:58				
Module 6: Junk Food	0:10:14	Module 6 - Plant foods: legumes	0:04:25				
		Module 7: Plant foods: nuts and seeds	0:03:54				
		Module 8: Plant foods: Grains	0:04:09				
		Module 9: Olive oil	0:03:42				
		Module 10: Fish and seafood	0:03:33				
		Module 11: Herbs and spices	0:03:16				
		Module 12: Alcohol	0:02:15				
		Module 13: Other food groups (red meat, dairy)	0:03:20				
		Module 14: Extras / Discretionary Foods	0:02:17				
		Module 15. Fermented foods	0:02:26				
		Module 16: The Mediterranean lifestyle	0:02:27				
**Total**	**0:56:38**		**0:52:05**		**0:52:40**		**0:52:40**
Activities - Goal Setting							
Goal Setting 1	0:01:00	Module 6 - Set a SMART goal	0:01:00	Week 3 SMART Goal	0:01:00	Goal Entries	0:06:00
Goal Setting 2	0:01:00	Module 7 - Set a SMART goal	0:01:00	Week 4 SMART Goal	0:01:00		
Goal Setting 3	0:01:00	Module 8 - Set a SMART goal	0:01:00	Week 5 SMART Goal	0:01:00		
Goal Setting 4	0:01:00	Module 9 - Set a SMART goal	0:01:00	Week 6 SMART Goal	0:01:00		
Goal Setting 5	0:01:00	Module 16 - Set a SMART goal	0:01:00	Week 7 SMART Goal	0:01:00		
Goal Setting 6	0:01:00	Module 11 - Set a SMART goal	0:01:00	Week 8 SMART Goal	0:01:00		
**Total**	**0:06:00**		**0:06:00**		**0:06:00**		**0:06:00**

Supplementary Material

My Food Mood Programs Key Activity Time Requirements

**My Food & Mood Version 1.0**		**My Food & Mood Version 2.1**		**My Food & Mood Version 2.2**		**My Food & Mood Version 3.0**	
Activities - Self Monitoring							
Self-Monitoring Food Entries	0:28:00	ModiMed Diet Score Activity	0:28:00	ModiMed Diet Score Activity	0:28:00	Food Entires	0:28:00
Self-Monitoring Mood Entires	0:14:00	Mood Check-ins	0:08:00			Mood Entires	0:10:00
**Total**	**0:42:00**	**Total**	**0:36:00**	**Total**	**0:28:00**	**Total**	**0:44:00**
Activities - Knowledge Verification & Skill building							
Health & Lifestyle Diet	0:02:00	Health & Lifestyle Diet	0:02:00	Health & Lifestyle Diet	0:02:00	Health & Lifestyle Diet	0:02:00
Interactive Mediterranean Diet	0:02:00	Activity: Can you spot a SMART Goal?	0:05:00	Setting SMART Goals	0:05:00	Shopping Lists	0:10:00
Fibre game	0:02:00	Review Quiz 1	0:05:00	Modules 1-8 Quiz	0:05:00	Modules 1-8 Quiz	0:05:00
Making Pre & Probiotics	0:05:00	Review Quiz 2	0:05:00	Modules 9-16 Quiz	0:05:00	Modules 9-16 Quiz	0:05:00
Seafood based recipe activity	0:05:00	Recipe Review	0:10:00	Recipe Review	0:20:00		
Snack building	0:02:00						
**Total**	**0:18:00**		**0:27:00**		**0:37:00**		**0:22:00**
**Total Time Key Activities**	**2:02:38**		**2:01:05**		**2:03:40**		**2:04:40**
