# Supplementary material for: Optimizing Engagement in an Online Dietary Intervention for Depression (My Food & Mood Version 3.0): Cohort Study
Source: JMIR Ment Health. 2021 Mar 31;8(3):e24871. doi: 10.2196/24871 (PMC8047812; doi:10.2196/24871)
Supplement: Multimedia Appendix 3 [file mental_v8i3e24871_app3.pdf]

My Food & Mood Program Screenshots

My Food & Mood Desktop Programs Login Screen

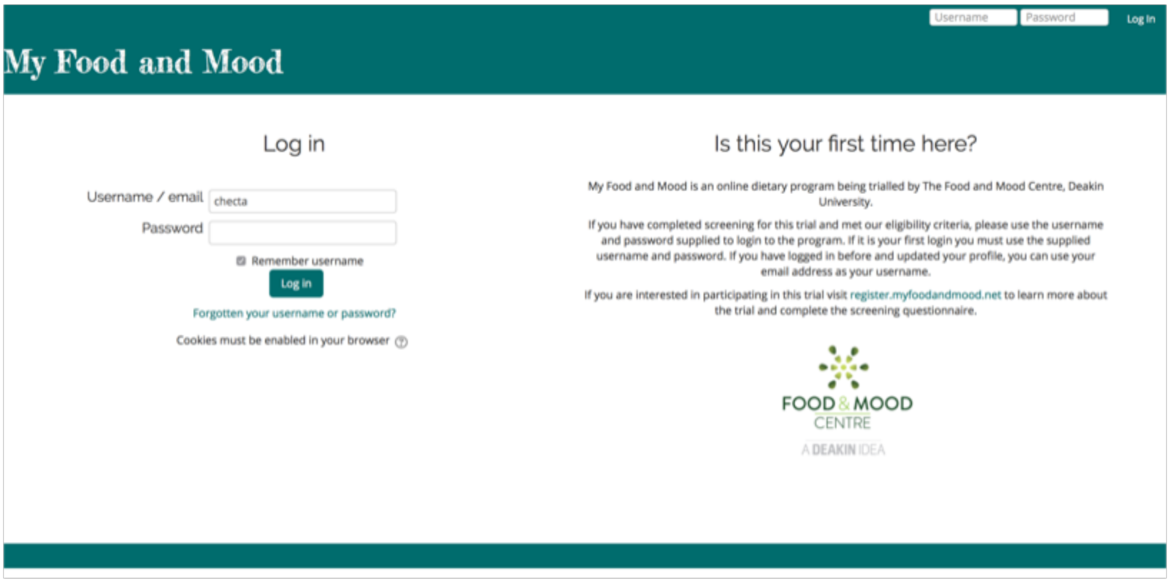

Figure 1: My Food and Mood Program desktop programs login screen

Version 1

Desktop Program

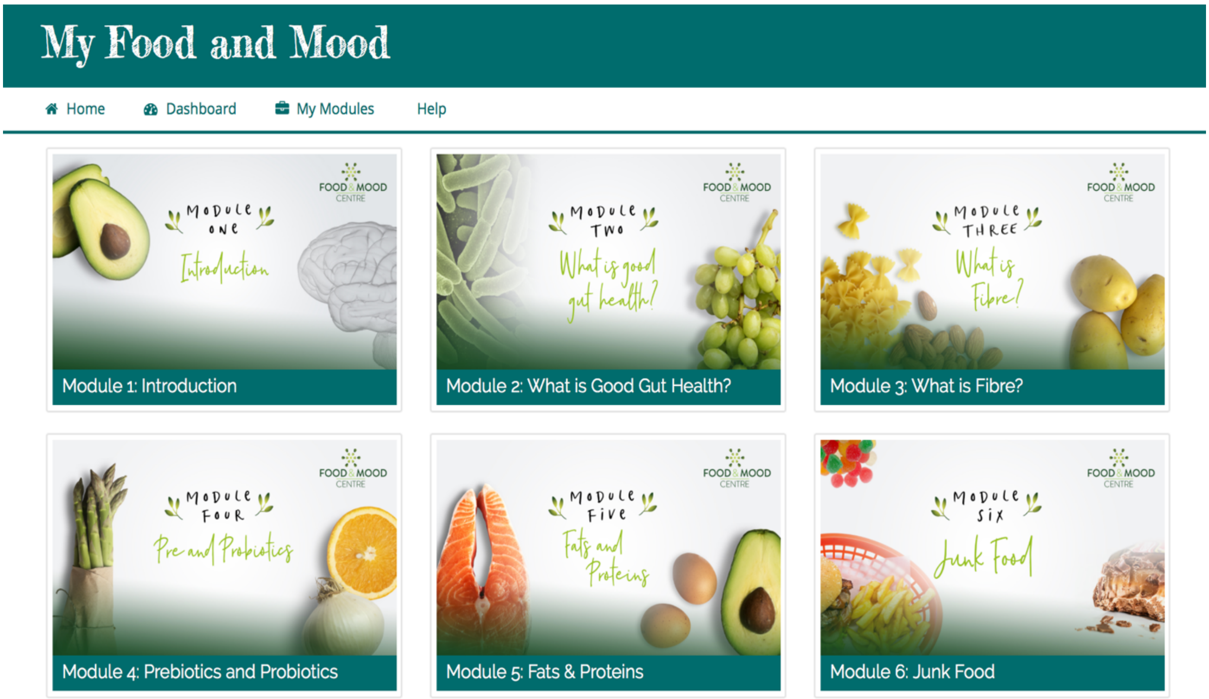

Figure 2: Screenshot of the My Food and Mood Program v1.0 Desktop program showing the program home page and 6 modules

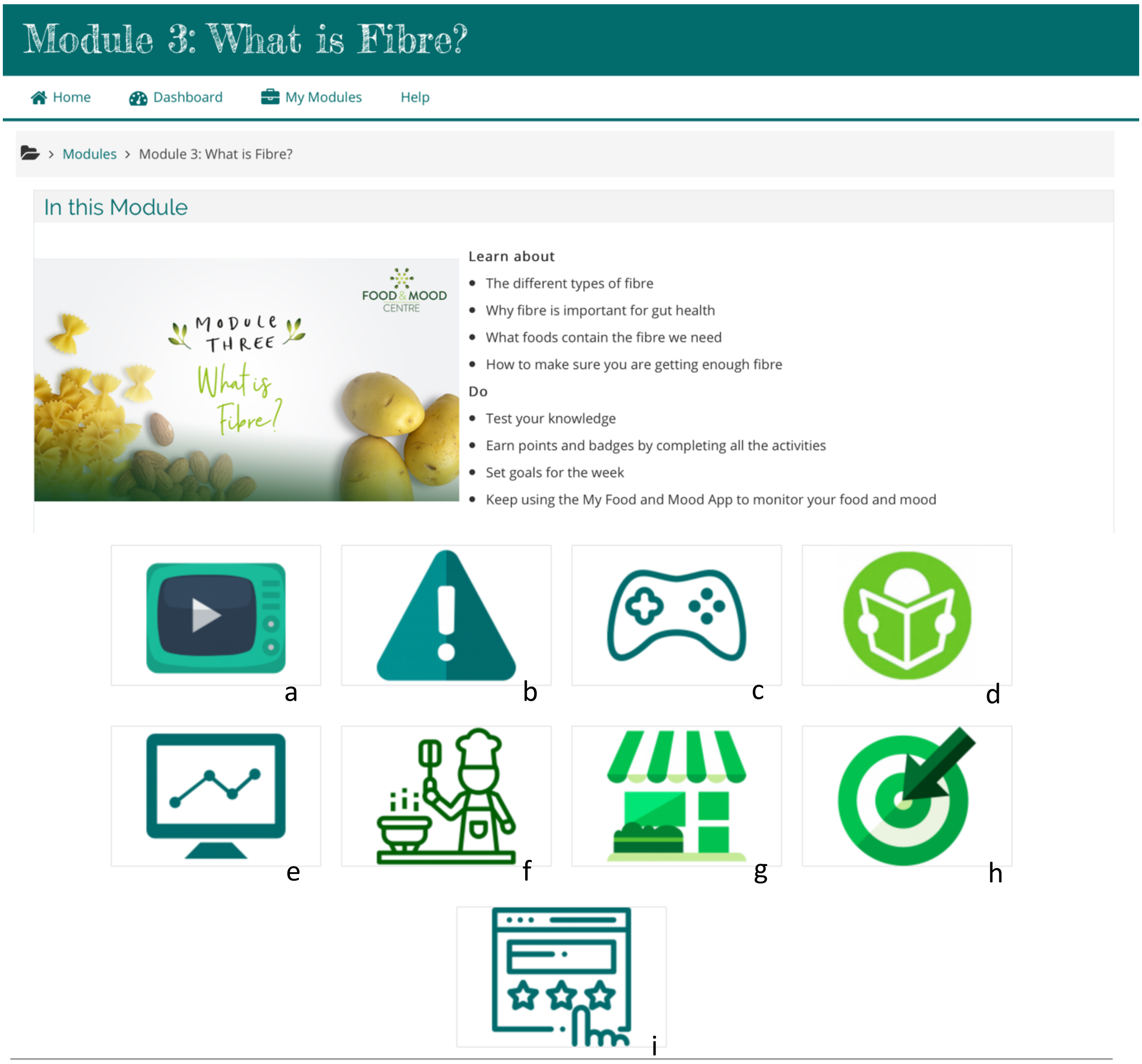

Figure 3: Screenshot from the My Food and Mood Program Version 1, showing the Module 3 home page and standard module layout with icons indicating the module activities (a) Watch the Video (b) Key Messages (c) Game (d) Reading (e) Progress (review your food & mood graph) (f) Recipes (g) Shopping list template (h) Goals (i) Module feedback

User Dashboard

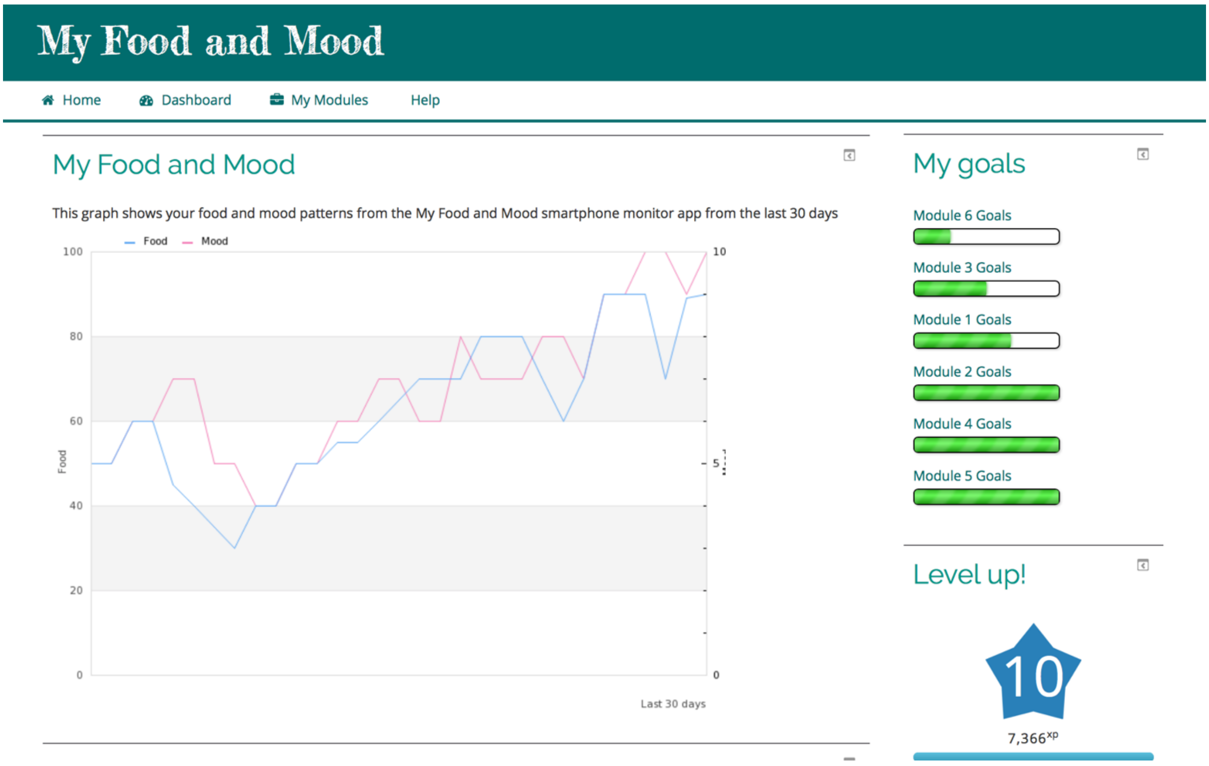

Figure 4: Screenshot of the My Food and Mood Program v1.0 Desktop program showing the program home page and 6 modules

Smartphone Monitoring Application

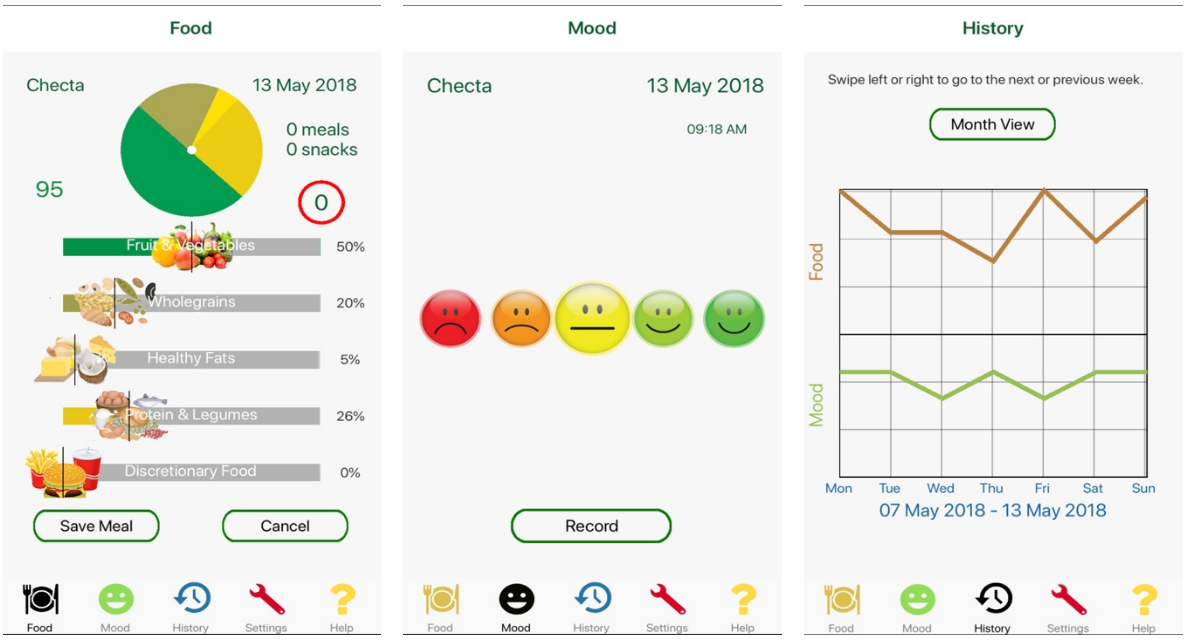

(a) (b) (c)

Figure 5: Screenshot of the My Food and Mood Program v1.0 Smartphone Monitoring application, showing the (a) Food data entry screen, (b) Mood data entry screen and (c) Food & Mood graph

Version 2

Desktop Programs

User Dashboard

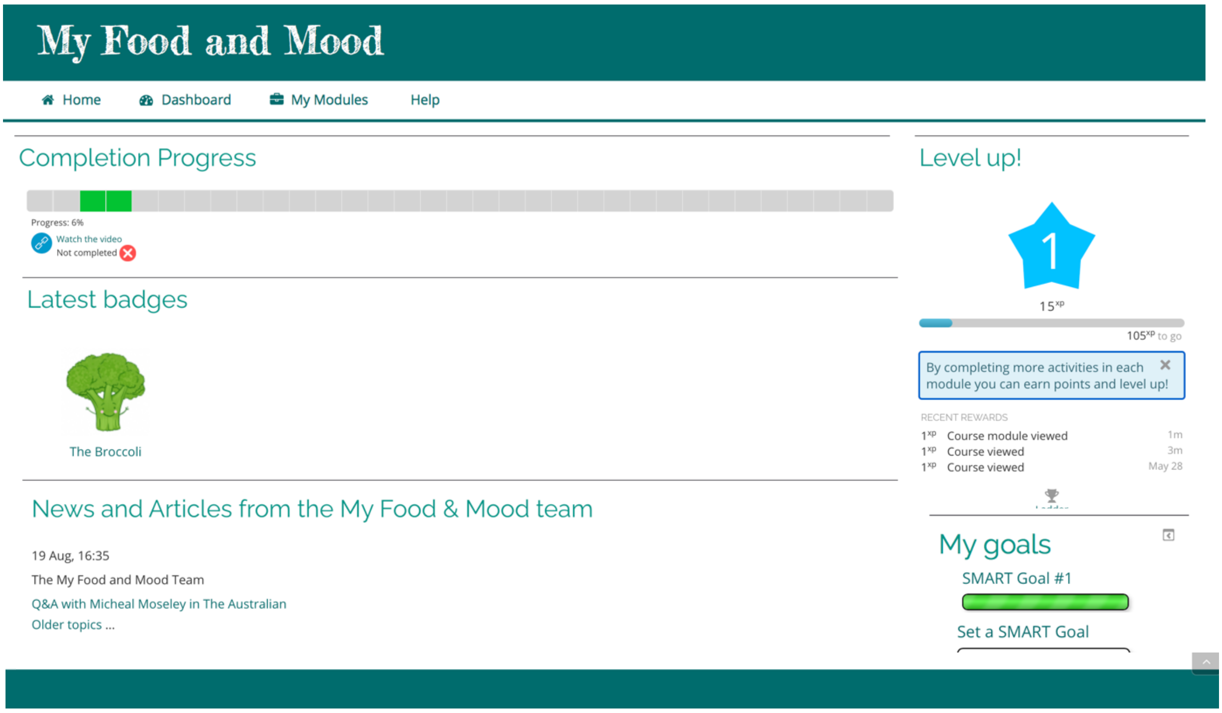

Figure 6: Screenshot of the My Food and Mood Program v1.0 Desktop program showing the program home page and 6 modules

My Modules Page

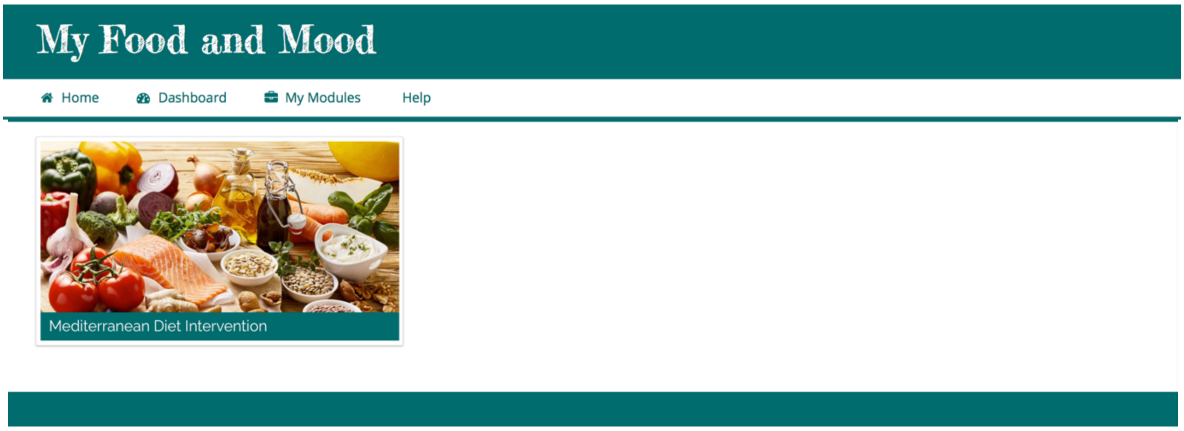

Figure 7: Screenshot of the My Food and Mood Program v1.0 Desktop program showing the program home page and 6 modules

Version 2.1

Program Page

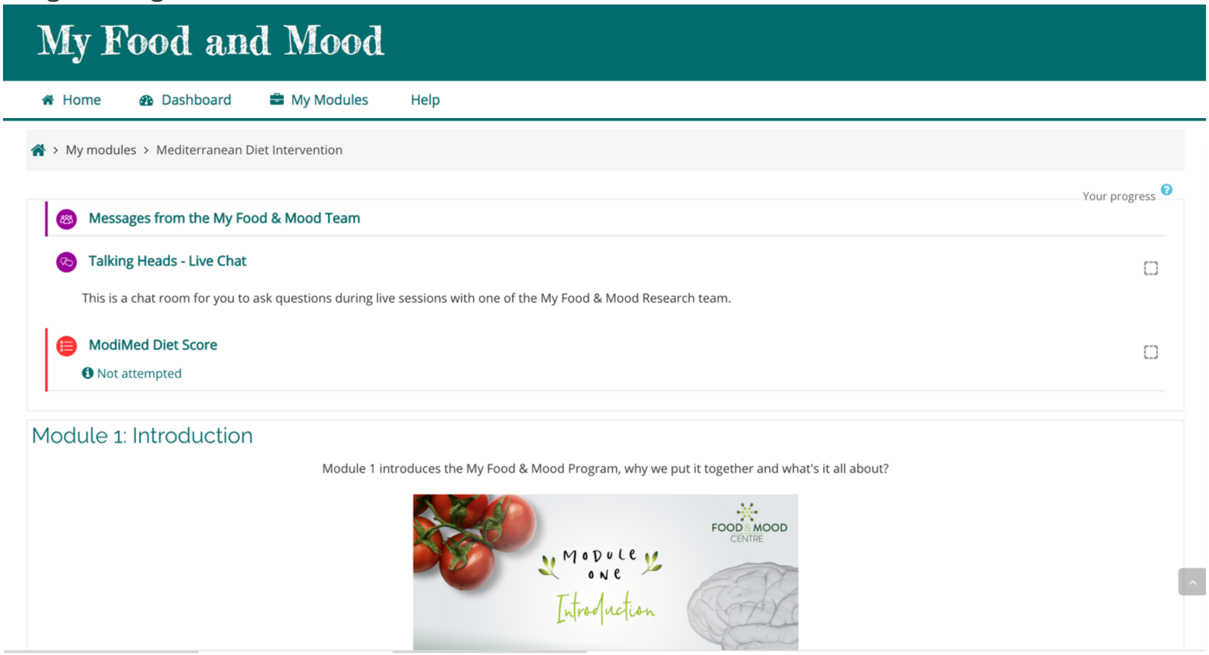

Figure 8: Screenshot of the My Food and Mood Program v2.1 Desktop program showing the program landing page with links to (a) Messages from the research team, (c) Live chat (b) diet tracking tool, and (d) Module 1

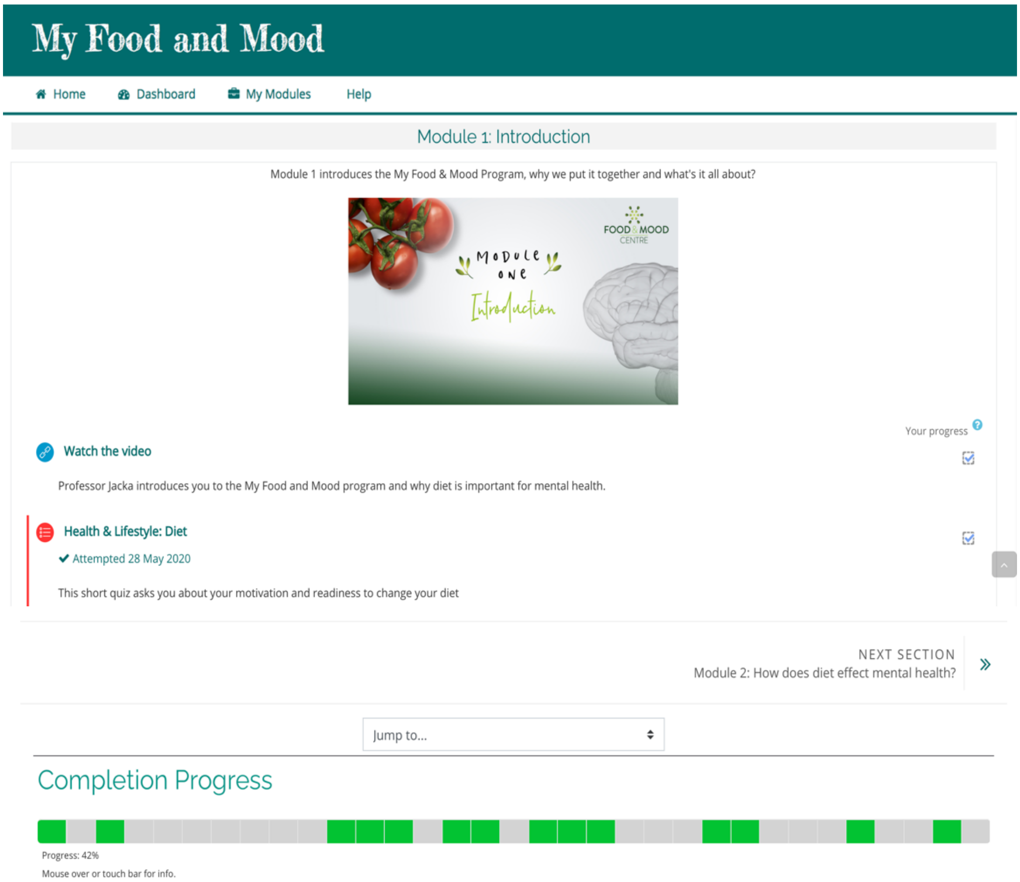

Figure 9: Screenshot of the My Food and Mood Program v2.1 Desktop program showing an example of a module page from Module 1 with activities and progress indicators

Version 2.2

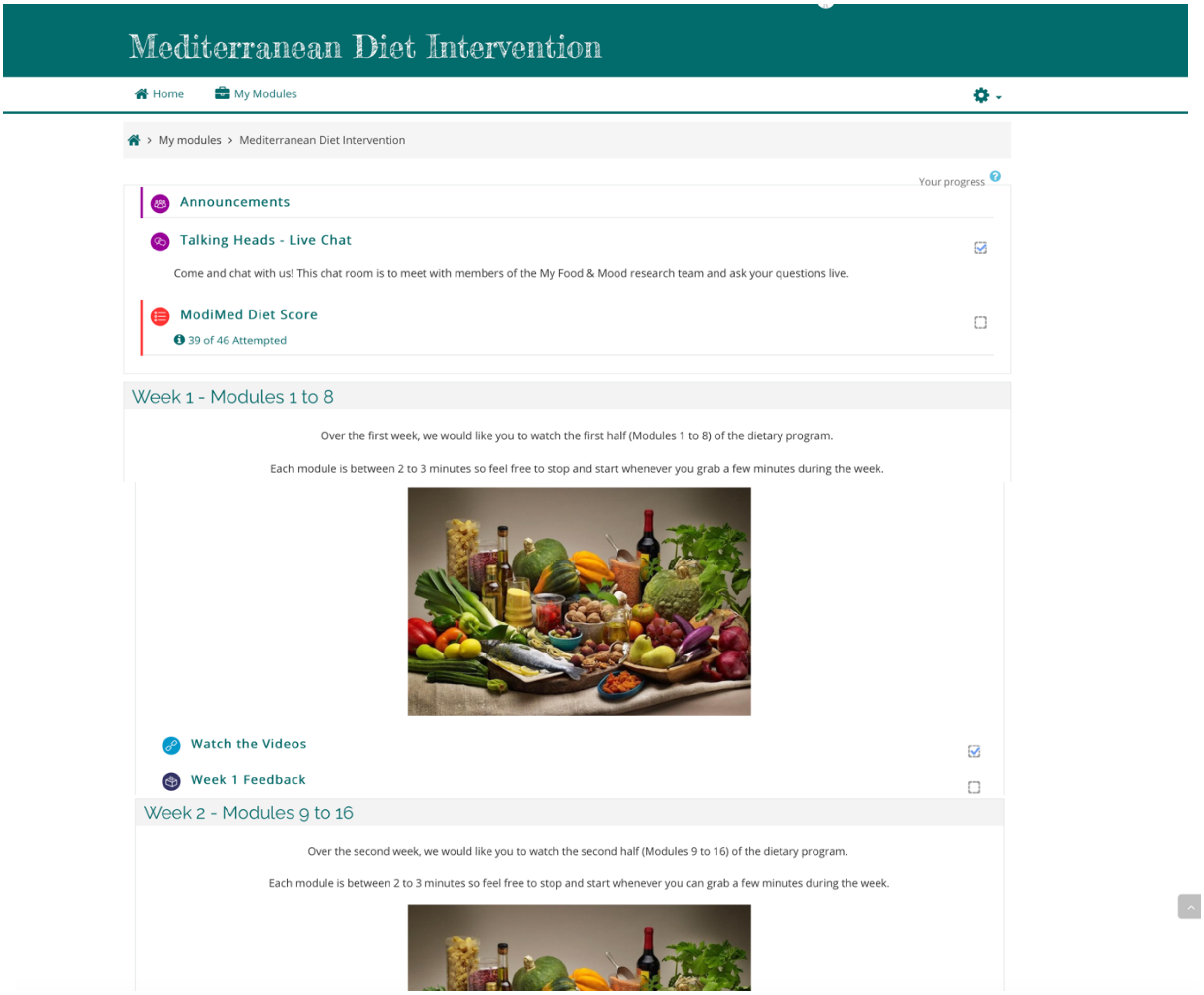

Figure 10: Screenshot of the My Food and Mood Program v2.2 Desktop program showing the an example of a module page from Module 1

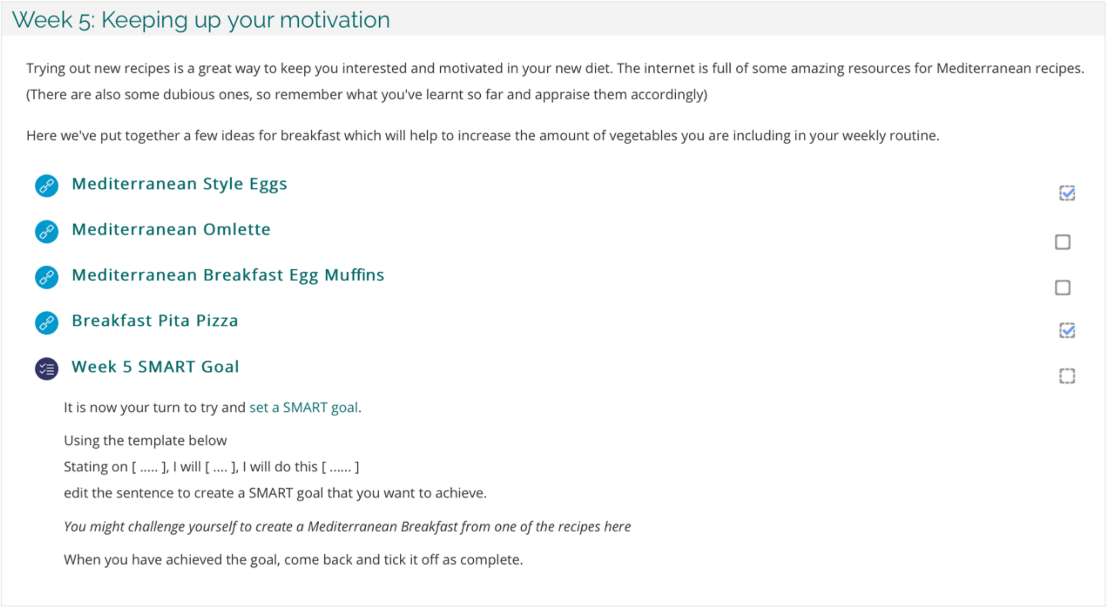

Figure 11: Screenshot of the My Food and Mood Program v2.2 Desktop program showing an example of one of Week 5 activities

Version 3

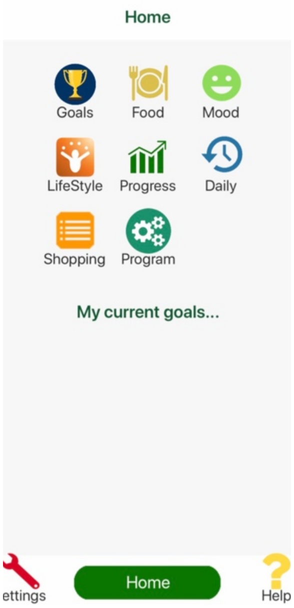

(a) Home Screen

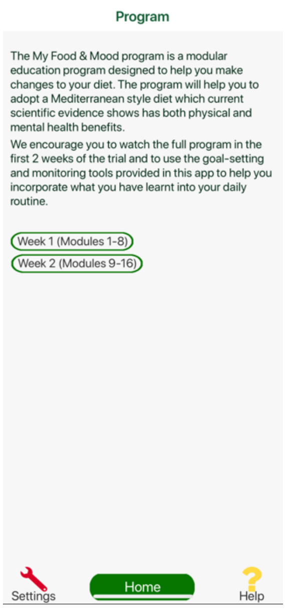

(b) Program screen (Video modules)

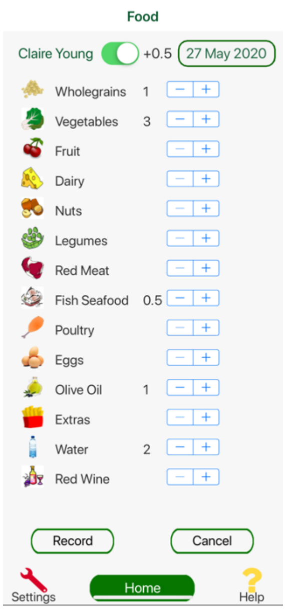

(c) Food data entry

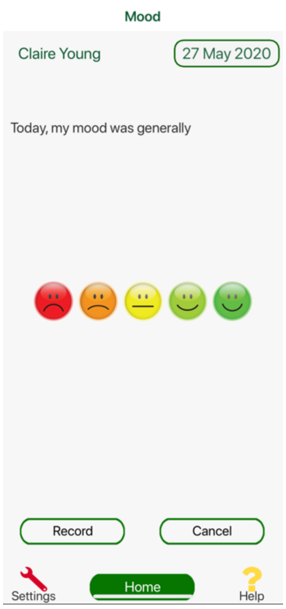

(d) Mood data entry

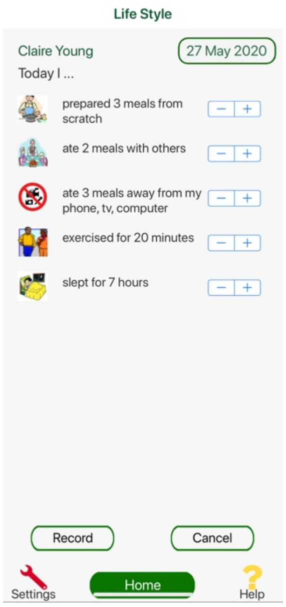

(e) Lifestyle data entry screen

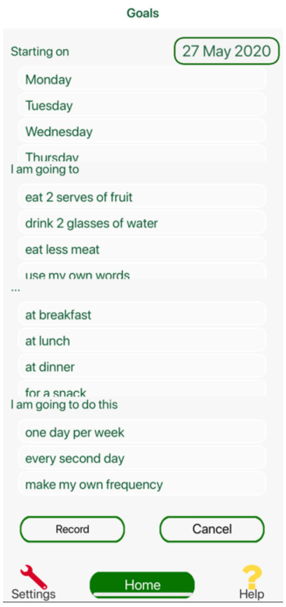

(f) Goals data entry screen

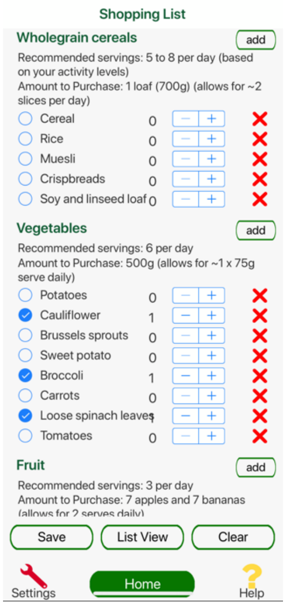

(f) Shopping list template screen

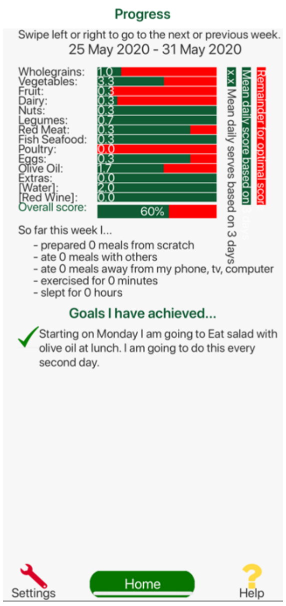

(g) Progress screen

Figure 12: My Food & Mood. Program Version 3.0 Screenshots
