# Supplementary material for: Optimizing Engagement in an Online Dietary Intervention for Depression (My Food & Mood Version 3.0): Cohort Study
Source: JMIR Ment Health. 2021 Mar 31;8(3):e24871. doi: 10.2196/24871 (PMC8047812; doi:10.2196/24871)
Supplement: Multimedia Appendix 4 [file mental_v8i3e24871_app4.pdf]

Supplementary material

Table 1: Mapping of SDQ and MEDAS questions used to calculate the MEDAS Rescored

MEDAS Questions	MEDAS Scoring	Mapped SDQ Questions	MEDAS Rescored
Q1. Do you use olive oil as the principal source of fat for cooking?	1 point for Yes	N/A	Not included
Q2. How much olive oil do you consume per day?	1 point for 4+ tablespoons per day	N/A	Not included
Q3. How many servings of vegetables do you consume per day?	1 point for 5+ serves per day	Q1. How many serves of vegetables do you usually eat each day?	1 point for 5+ serves per day
Q4. How many pieces of fruit do you consume per day?	1 point for 3+ serves per day	Q3. How many serves of fruit do you usually eat each day?	1 point for 3+ serves per day
Q5. How many servings of red meat, hamburger, or sausages do you consume per day?	1 point for <1 serve per day	23. How often do you usually eat meat or chicken?<br>24. Which of these meats have you eaten in the last 2 weeks? Tick all that apply.	1 point for <1 serve per day<br>*If red meats were selected in Q24, the percentage of options that were red meat was calculated and applied to serves in Q23, If no red meat selected in Q24, then 1 point given*
Q6. How many servings (12 g) of butter, margarine, or cream do you consume per day?	1 point for 3+ serves per day	N/A	Not included
Q7. How many carbonated and/or sugar-sweetened beverages do you consume per day?	1 point for None	Q16. How often do you drink soft drinks, cordial, fruit or sports drinks?	1 point for <1 serve per day

MEDAS Questions	MEDAS Scoring	Mapped SDQ Questions	MEDAS Rescored
Q8. Do you drink red wine? How much do you consume per week?	1 point for 7+ standard drinks	N/A	Not included
Q9. How many servings of pulses do you consume per week?	1 point for 4+ serves per week	Q19. How often do you usually eat legumes and soy products (e.g. peas, beans, lentils, tofu)?	1 point for 4+ serves per week<br>*Every second day and more frequent*
Q10. How many servings of fish/seafood do you consume per week?	1 point for 3+ serves per week	Q21. How often do you usually eat fresh or canned fish?	1 point for 3+ serves per week<br>*Every second day and more frequent*
Q11. How many times do you consume commercial (not homemade) pastry such as cookies or cake per week?	1 point for <3 serves per week	Q12. How often do you eat snacks such as packet potato chips, biscuits (sweet or savoury), chocolate, lollies, cakes and sweet pastry?	1 point for <3 serves per week<br>*Twice a week and less frequent*
Q12. How many times do you consume nuts per week?	1 point for 3+ serves per week	14. How often do you usually eat nuts?	1 point for 3+ serves per week<br>*Every second day and more frequent*
Q13. Do you prefer to eat chicken, turkey or rabbit instead of beef, pork, hamburgers, or sausages?	1 point for “Yes” or “Vegetarian”	Q23 How often do you usually eat meat or chicken?<br>Q24 Which of these meats have you eaten in the last 2 weeks? Tick all that apply.	1 point if Q23 is None<br><br>1 point if *the percentage of options selected in Q24 for poultry is larger than red meat.*
Q14. How many times per week do you consume boiled vegetables, pasta, rice, or other dishes with a sauce of tomato, garlic, onion, or leeks sautéed in olive oil?	1 point for 3+ serves per week	N/A	Not included

MEDAS Questions	MEDAS Scoring	Mapped SDQ Questions	MEDAS Rescored
Total Points	14		9
