# Supplementary material for: Optimizing Engagement in an Online Dietary Intervention for Depression (My Food & Mood Version 3.0): Cohort Study
Source: JMIR Ment Health. 2021 Mar 31;8(3):e24871. doi: 10.2196/24871 (PMC8047812; doi:10.2196/24871)
Supplement: Multimedia Appendix 5 [file mental_v8i3e24871_app5.pdf]

My Food & Mood Project (Cohort 1)

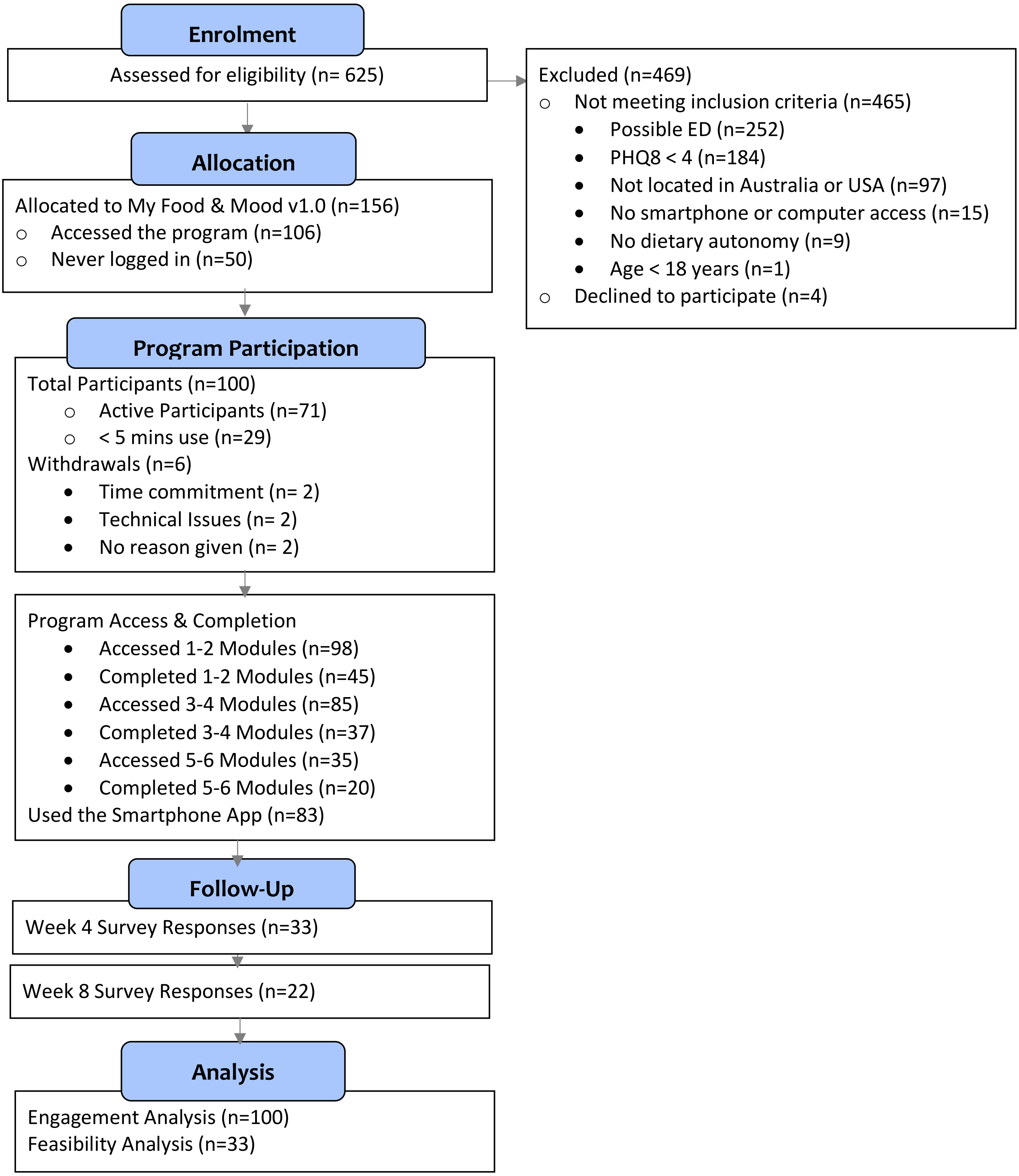

Figure 1: CONSORT Diagram for the My Food & Mood Project (Cohort 1)

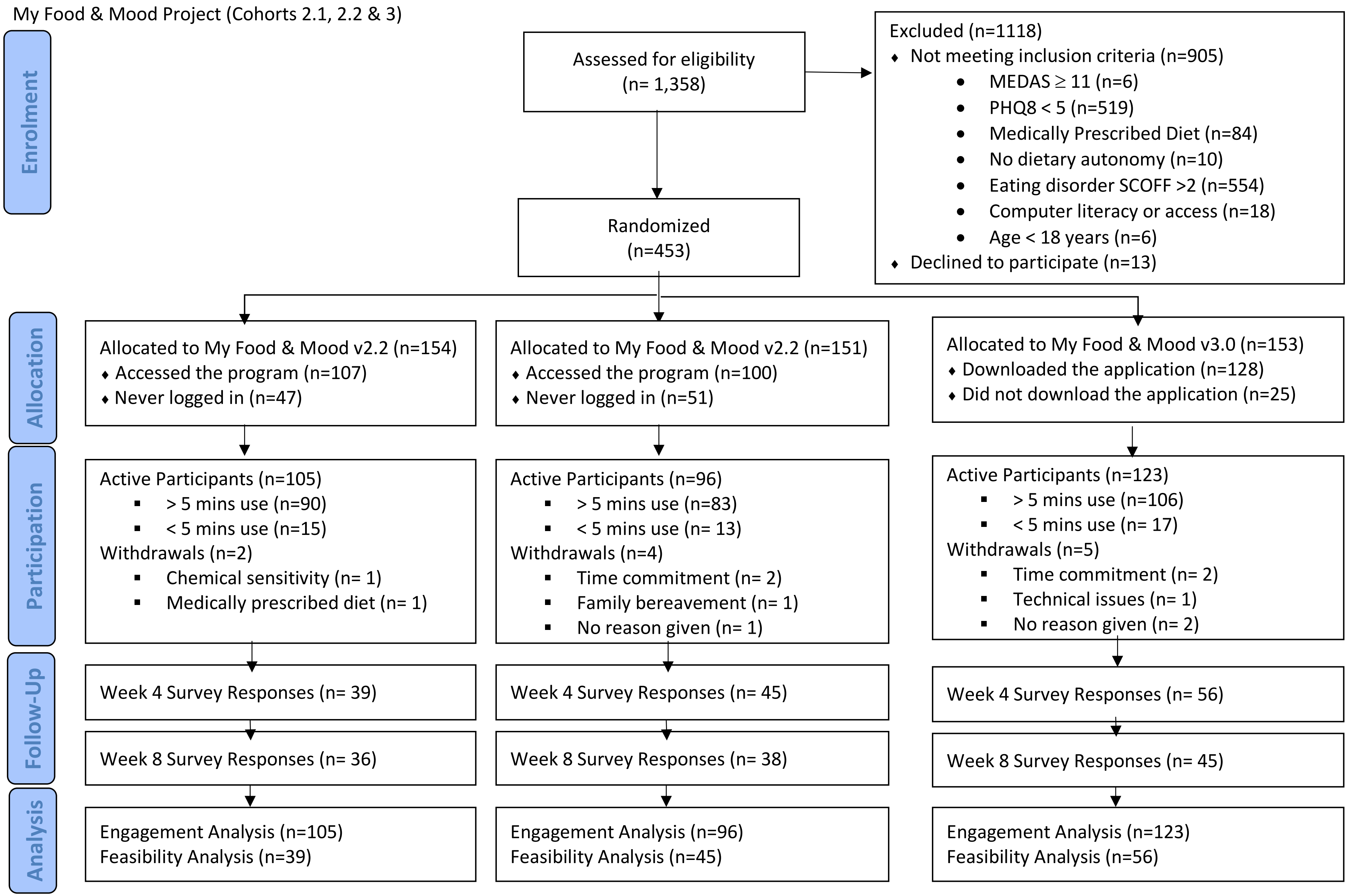

Figure 2: CONSORT Diagram for the My Food & Mood Project (Cohorts 2.1, 2.2 & 3.0)
